# The Impact of HAART on the Respiratory Complications of HIV Infection: Longitudinal Trends in the MACS and WIHS Cohorts

**DOI:** 10.1371/journal.pone.0058812

**Published:** 2013-03-12

**Authors:** Matthew R. Gingo, G. K. Balasubramani, Lawrence Kingsley, Charles R. Rinaldo, Christine B. Alden, Roger Detels, Ruth M. Greenblatt, Nancy A. Hessol, Susan Holman, Laurence Huang, Eric C. Kleerup, John Phair, Sarah H. Sutton, Eric C. Seaberg, Joseph B. Margolick, Stephen R. Wisniewski, Alison Morris

**Affiliations:** 1 Department of Medicine, School of Medicine, University of Pittsburgh, Pittsburgh, Pennsylvania, United States of America; 2 Department of Epidemiology, School of Public Health, University of Pittsburgh, Pittsburgh, Pennsylvania, United States of America; 3 Department of Infectious Diseases and Microbiology, School of Public Health, University of Pittsburgh, Pittsburgh, Pennsylvania, United States of America; 4 Department of Pathology, School of Medicine, University of Pittsburgh, Pittsburgh, Pennsylvania, United States of America; 5 WIHS Data Management and Analysis Center, Department of Epidemiology, The Johns Hopkins University Bloomberg School of Public Health, Baltimore, Maryland, United States of America; 6 Department of Epidemiology, School of Public Health, University of California Los Angeles, Los Angeles, California, United States of America; 7 Department of Clinical Pharmacy, School of Pharmacy, University of California San Francisco, San Francisco, California, United States of America; 8 Department of Medicine, School of Medicine, University of California San Francisco, San Francisco, California, United States of America; 9 Department of Epidemiology and Biostatistics, School of Medicine, University of California San Francisco, San Francisco, California, United States of America; 10 Department of Medicine, SUNY Downstate Medical Center, Brooklyn, New York, United States of America; 11 Department of Medicine, David Geffen School of Medicine, University of California San Francisco, San Francisco, California, United States of America; 12 Department of Medicine, Feinberg School of Medicine, Northwestern University, Chicago, Illinois, United States of America; 13 Department of Epidemiology, Bloomberg School of Public Health, Johns Hopkins University, Baltimore, Maryland, United States of America; 14 Department of Molecular Microbiology and Immunology, Bloomberg School of Public Health, Johns Hopkins University, Baltimore, Maryland, United States of America; 15 Department of Immunology, School of Medicine, University of Pittsburgh, Pittsburgh, Pennsylvania, United States of America; Infectious Disease Service, United States of America

## Abstract

**Objective:**

To review the incidence of respiratory conditions and their effect on mortality in HIV-infected and uninfected individuals prior to and during the era of highly active antiretroviral therapy (HAART).

**Design:**

Two large observational cohorts of HIV-infected and HIV-uninfected men (Multicenter AIDS Cohort Study [MACS]) and women (Women’s Interagency HIV Study [WIHS]), followed since 1984 and 1994, respectively.

**Methods:**

Adjusted odds or hazards ratios for incident respiratory infections or non-infectious respiratory diagnoses, respectively, in HIV-infected compared to HIV-uninfected individuals in both the pre-HAART (MACS only) and HAART eras; and adjusted Cox proportional hazard ratios for mortality in HIV-infected persons with lung disease during the HAART era.

**Results:**

Compared to HIV-uninfected participants, HIV-infected individuals had more incident respiratory infections both pre-HAART (MACS, odds ratio [adjusted-OR], 2.4; 95% confidence interval [CI], 2.2–2.7; p<0.001) and after HAART availability (MACS, adjusted-OR, 1.5; 95%CI 1.3–1.7; p<0.001; WIHS adjusted-OR, 2.2; 95%CI 1.8–2.7; p<0.001). Chronic obstructive pulmonary disease was more common in MACS HIV-infected vs. HIV-uninfected participants pre-HAART (hazard ratio [adjusted-HR] 2.9; 95%CI, 1.02–8.4; p = 0.046). After HAART availability, non-infectious lung diseases were not significantly more common in HIV-infected participants in either MACS or WIHS participants. HIV-infected participants in the HAART era with respiratory infections had an increased risk of death compared to those without infections (MACS adjusted-HR, 1.5; 95%CI, 1.3–1.7; p<0.001; WIHS adjusted-HR, 1.9; 95%CI, 1.5–2.4; p<0.001).

**Conclusion:**

HIV infection remained a significant risk for infectious respiratory diseases after the introduction of HAART, and infectious respiratory diseases were associated with an increased risk of mortality.

## Introduction

The incidence of respiratory diseases and their effect on survival in the era of highly active antiretroviral therapy (HAART) are largely unknown. Few studies have examined changes in respiratory diseases incidence spanning different treatment eras of the Human Immunodeficiency Virus (HIV) epidemic. Infectious respiratory complications of HIV infection have historically been a major cause of morbidity and mortality in the HIV-infected population[1]–[4]. Although infectious lung diseases have decreased after HAART, they still occur, and the impact of HAART on non-infectious lung diseases, such as obstructive pulmonary disease[5]–[7], bronchogenic carcinoma[8]–[10], pulmonary hypertension [11], [12], and pulmonary fibrosis [6] is less clear.

The Pulmonary Complications of HIV Study (PCHIS), spanning 1988–1994, demonstrated that HIV-infected individuals had higher rates of upper and lower respiratory infections than HIV-uninfected participants [3]. The PCHIS generated much of the information known about HIV-associated pulmonary diseases and significantly impacted patient care. The study reflected the HIV/AIDS population early in the HIV epidemic, with most participants being men who had sex with men, and stopped prior to the availability of HAART. Since the PCHIS study, epidemiology and treatment of HIV have drastically changed. More women, minorities, and intravenous drug users are now HIV-infected; and receipt of HAART is the leading predictor of survival in HIV infection[1], [13]–[17]. Epidemiologic and therapeutic changes with respect to HIV infection in the United States severely limit our ability to generalize the findings of the PCHIS and similar studies to the current environment.

To address this question, we have reviewed the incidence of respiratory conditions and their effect on mortality in the era of HAART in two large cohorts comparing HIV-infected and uninfected individuals.

## Methods

### Cohorts

The Multicenter AIDS Cohort Study (MACS) is an ongoing multicenter prospective cohort study started in 1984 at four centers across the United States, enrolling 6,972 HIV-infected or at-risk men who have sex with men [18]: 4,954 in 1984; 668 between 1987 and 1991; and 1,350 between 2001 and 2003. The Women’s Interagency HIV Study (WIHS) is an ongoing multicenter prospective cohort study from six centers across the United States which began in 1994 enrolling 3,766 women with or at risk for HIV [19]: 2,625 in 1994–1995 and 1,143 in 2001–2002. The institutional review boards of participating institutions (University of Pittsburgh; Women’s Interagency HIV Study Data Management and Analysis Center; Johns Hopkins University; University of California, Los Angeles; University of California, San Francisco; State University of New York Downstate Medical Center; Northwestern University; and the Center for Analysis and Management of Multicenter AIDS Cohort Study) approved the study protocol, and written informed consent was obtained from all participants.

### Data Collection

Baseline and 6-month follow-up visits consisted of interviewer-administered, structured assessments and specimen collection. Infectious diagnoses confirmed by medical record review with standardized criteria in each cohort included *Pneumocystis* pneumonia (PCP), bacterial pneumonia, non-tuberculosis mycobacterial infection (NTM), and pulmonary tuberculosis (TB) per cohort protocols [18], [19]. Infectious diagnoses obtained from participant self-report were acute sinusitis and acute bronchitis in both MACS and WIHS by asking at each six-month visit if they had had an episode of acute sinusitis or bronchitis. Self-reported lung cancer cases were confirmed for both cohorts by either searches of statewide cancer registries or medical record confirmation. Pulmonary Kaposi sarcoma was not included in lung cancer diagnoses. Participant self-report of chronic obstructive pulmonary disease (COPD) in MACS and asthma in WIHS were also recorded. Participant characteristics and clinical data were prospectively collected and were extracted for current analysis either at baseline or at the appropriate study visit and included: age, race, ethnicity, smoking history, alcohol use, illicit drug use, antiretroviral drug use, opportunistic infection prophylaxis, CD4 lymphocyte counts, HIV serostatus, and plasma HIV RNA quantification. Self-reports of smoking at each visit were used to determine smoking status (current, former, or never smokers) and pack-year history of smoking. Alcohol use was determined for each visit and classified into none, light drinking (<3 drinks/week), moderate drinking (3–13 drinks/week), and heavy drinking (≥14 drinks/week). Classification of illicit drug use was based on participant self-report data at each visit for use of marijuana, crack, cocaine, heroin, methamphetamines, poppers (inhaled alkyl nitrites), and other drugs. Participants were classified as having used illicit or intravenous drugs during the six months prior to their visit by self-report. HAART was classified as use of combination antiretroviral therapy as previously described [20].

### Statistical Methods

Data were analyzed with SAS v9.2 (SAS Institute, Inc., Cary, NC). We analyzed respiratory diseases data collected in the pre-HAART (from 1984 until October 31, 1994) and HAART (January 1, 1996 until data were frozen for analysis on December 31, 2008) eras. Outcomes included infectious and non-infectious respiratory diseases and all-cause mortality. Incidence rates were computed as number of observed incident events divided by number of person-years of follow-up, where follow-up time available for each person was the number of years from baseline visit until the earliest of the disease diagnosis date (for non-infectious diagnoses), death, loss to follow-up, or the date of the last study visit on or prior to December 31, 2008. An infectious outcome was defined as the presence of any of the following: PCP, bacterial pneumonia, TB, NTM, acute sinusitis, or acute bronchitis. A non-infectious outcome was defined as the presence of lung cancer or COPD for MACS and lung cancer or asthma for WIHS. For infectious diagnoses, repeated events in a single participant were considered in the analysis. Once a participant had a non-infectious respiratory diagnosis, data from further visits were excluded from analyses of that diagnosis, but were included in analyses of other non-infectious or infectious diagnoses. Incidence data were determined separately for participants in MACS pre-HAART and HAART periods, and in WIHS for the HAART period only. The cohorts were analyzed separately because of differences in the populations sampled such as gender, proportions of minorities, socioeconomic status, access to health care, and substance use as well as variations in data collection procedures. Only the MACS had sufficient duration of data collection during the pre-HAART era for analysis.

Summary statistics of demographics, social, and clinical characteristics were calculated for the MACS (pre-HAART and HAART) and WIHS (HAART) for those with HIV and without HIV. To evaluate the association between infectious respiratory diseases and HIV status for the pre-HAART and HAART eras, we fit separate multivariate models and analyzed longitudinally with Generalized Estimating Equations (GEE) [21], [22]. The outcome variable is the odds of a respiratory illness and the unit of analyses are six month person visit. GEE method was used to account for multiple observations within a patient.

To evaluate the association between non-infectious respiratory diseases and HIV status for the pre-HAART and HAART eras, the Cox’s proportional hazard model was used.Cox-proportional regression methods were used to assess all-cause mortality related to having any infectious or non-infectious respiratory disease, adjusting for age, race, pack-years smoking, intravenous drug use, and alcohol use. Data were censored at date of death or, if alive, date of last follow-up visit. Models considered covariates time-variant (age, pack-years smoking, intravenous drug use, and alcohol use) and included both any infectious outcome and any non-infectious outcome, excluding events that occurred within one year prior to death. Cox models considering the covariates at the time of origin of the survival period were also performed and had similar results as the models considering covariates as time-variant. Only the results of the models with time-variant covariates are reported. The main predictor, respiratory illnesses, in the mortality models were accounted as the occurrence of the first ever event. The time of origin for all Cox models are from the beginning of the post HAART era (i.e. From January 1, 1996). Cox models performed with and without including acute sinusitis and bronchitis (infections not likely associated with mortality) did not differ in interpretation, and only models including sinusitis and bronchitis are reported.

Adjusted odds ratios (adjusted-OR) or hazards ratios (adjusted-HR) and 95% confidence intervals (CI) are reported unless otherwise noted. An alpha level of 0.05 was used to indicate statistical significance.

## Results

### Cohort Characteristics

There were 2,799 HIV-infected men and 2,821 HIV-uninfected men from the MACS cohort who were enrolled and followed during the pre-HAART era. For the HAART era, there were 1,706 HIV-infected and 1,774 HIV-uninfected men from the MACS cohort, and for the WIHS cohort, there were 2,507 HIV-infected and 879 HIV-uninfected females. Person-years of follow-up are reported in **Tables 1** **and** **2**. Median years of follow-up ranged from 5.5 to 9.5. In MACS, the baseline characteristics age, race, ethnicity, smoking status and pack-years of smoking, alcohol use, and intravenous and illicit drug use differed between HIV-infected and -uninfected participants in both the pre-HAART and HAART eras (**Table 1**). The WIHS participants in the HAART era were more evenly matched, but HIV-infected participants differed from HIV-uninfected participants in age, pack-years of smoking, alcohol use, and illicit drug use (**Table 2**).

**Table 1:** MACS participant characteristics.

			MACS Pre-HAART			MACS HAART era		
			HIV−	HIV+		HIV−	HIV+	
N			2821	2799		1774	1706	
Follow-up, years; median (Q1–Q3)			9.5 (6.3–9.9)	6.4 (3.0–9.6)		6.5 (4.5–12.0)	8.5 (5.2–12.3)	
Age at baseline visit, years, mean (SD)			33.8 (8.3)	32.2 (6.9)	**<0.001**	34.8 (8.6)	34.1 (8.3)	**0.01**
Race, n (%)	White		2556 (90.6)	2387 (85.3)	**<0.001**	1293 (72.9)	1097 (64.3)	**<0.001**
	Black		223 (7.9)	376 (13.4)		403 (22.7)	480 (28.1)	
	Other		39 (1.4)	34 (1.2)		78 (4.4)	129 (7.6)	
Ethnicity, Hispanic, n (%)			110 (3.9)	189 (6.8)	**<0.001**	136 (7.7)	240 (14.1)	**<0.001**
Smoking status^a^, n (%)	Never		1227 (44.8)	1063 (39.4)	**<0.001**	394 (29.7)	326 (27.1)	0.17
	Former		431 (15.7)	367 (13.6)		421(31.8)	421 (35.0)	
	Current		1082 (39.5)	1265 (47.0)		511(38.5)	456 (37.9)	
Pack-years smoking (ever smokers), median (Q1–Q3)			17.7 (5.2–31.5)	15.6 (5.1–29.1)	**0.01**	8.2 (0.9–27)	12.8 (2–30.6)	**0.006**
Alcohol use^a^, n (%)	None		297 (11.5)	250 (9.6)	**<0.001**	376 (28.4)	431 (35.9)	**<0.001**
	Light		755 (29.3)	647 (24.9)		360 (27.1)	349 (29.1)	
	Moderate		1070 (41.5)	1161 (44.7)		439 (33.1)	328 (27.3)	
	Heavy		456 (17.7)	538 (20.7)		151 (11.4)	92 (7.7)	
Intravenous drug use, ever^a^, n (%)			109 (3.9)	372 (13.4)	**<0.001**	160 (9)	307 (18)	**<0.001**
Illicit drug use, ever^a^, n (%)			2310 (86.0)	2567 (94.7)	**<0.001**	1427 (81)	1432 (84.3)	**0.01**
*Pneumocystis* prophylaxis use, ever^a^, n (%)			–	1026 (46.9)	–	–	686 (40.2)	–
HAART use, ever, n (%)			–	–		–	1407 (82.5)	–
CD4 lymphocyte count, cells/µL, median (Q1–Q3)			893 (692–1160)	595 (423–826)	**<0.001**	912 (720–1144)	357 (193–536)	**<0.001**
Log_10_ HIV RNA level, copies/ml, mean (SD)			–	3.6 (1.4)	–	–	4.1 (1.1)	–
HIV RNA level <400 copies/ml, n (%)			–	–	–	–	1220 (76)	–
Visits with HIV RNA<400 copies/ml, median % (Q1–Q3)			–	–	–	–	70 (41–95)	

a Some variables do not sum to the total n due to missing data at baseline.

HAART = highly active antiretroviral therapy, SD = standard deviation, Q1 = quartile 1, Q3 = quartile 3.

**Table 2:** WIHS participant characteristics.

			WIHS HAART era		
			HIV-	HIV+	
N			879	2507	
Follow-up, years; median (Q1–Q3)			5.5 (4.9–11.0)	5.7 (4.6–11.1)	
Age at baseline visit, years, mean (SD)			31.9 (8.8)	35.3 (7.8)	**<0.001**
Race, n (%)	White		211 (24.0)	578 (23.1)	0.83
	Black		505 (57.5)	1464 (58.4)	
	Other		159 (18.1)	459 (18.3)	
Ethnicity, Hispanic, n (%)			243 (27.7)	653 (26.0)	0.34
Smoking status^a^, n (%)	Never		268 (30.6)	791 (31.8)	0.32
	Former		136 (15.6)	427 (17.2)	
	Current		471 (53.8)	1270 (51.0)	
Pack-years smoking (ever smokers), median (Q1–Q3)			13 (6.5–21)	17 (10–23)	**<0.001**
Alcohol use^a^, n (%)	None		398 (46.0)	1387 (56.4)	**<0.001**
	Light		248 (28.7)	611 (24.8)	
	Moderate		143 (16.5)	304 (12.4)	
	Heavy		76 (8.8)	157 (6.4)	
Intravenous drug use, ever^a^, n (%)			105 (11.9)	1326 (13.0)	0.42
Illicit drug use, ever^a^, n (%)			576 (72.5)	1332 (62.2)	**<0.001**
*Pneumocystis* prophylaxis use, ever, n (%)			–	1698 (67.7)	–
HAART use, ever, n (%)			–	1963 (78.3)	–
CD4 lymphocyte count, cells/µL, median (Q1–Q3)			1008 (794–1257)	382 (211–581)	**<0.001**
Log HIV RNA level, copies/ml, mean (SD)			–	3.8 (1.1)	–
HIV RNA level <400 copies/ml, n (%)			–	1822 (73.3)	–
Visits with HIV RNA<400 copies/ml, median % (Q1–Q3)			–	47.3 (23.8–75)	–

a Some variables do not sum to the total n due to missing data at baseline.

HAART = highly active antiretroviral therapy, SD = standard deviation, Q1 = quartile 1, Q3 = quartile 3.

### Infectious Respiratory Diseases

HIV-infected individuals in MACS had greater risk of incident respiratory infections than HIV-uninfected participants in the pre-HAART era (**Table 3**). There were 30.59 cases of PCP, 0.22 cases of NTM, and 0.50 cases of TB per 1,000 person-years of follow-up in HIV-infected participants compared to no cases of these diseases in HIV-uninfected participants (**Table 4**). HIV-infected participants also had increased risk of bacterial pneumonia, acute sinusitis, acute bronchitis, and any infectious respiratory disease (i.e. PCP, NTM, TB, bacterial pneumonia, sinusitis, or bronchitis).

**Table 3:** Unadjusted and adjusted estimates of infectious and non-infectious complications in HIV-infected persons.

	Unadjusted		Adjusted	
MACS Pre-HAART	OR (95% CI)	p-value	OR (95% CI)	p–value
Infectious Diagnosis				
Bacterial pneumonia	21.9 (11.9–40.5)	<0.001	21.8 (11.7–40.6)	<0.001
Acute sinusitis	1.39 (1.24–1.55)	<0.001	1.64 (1.44–1.86)	<0.001
Acute bronchitis	1.70 (1.46–1.97)	<0.001	1.78 (1.48–2.13)	<0.001
Any infectious diagnosis	1.86 (1.71–2.03)	<0.001	2.43 (2.20–2.68)	<0.001
	HR (95% CI)	p–value	HR (95% CI)	p-value
Non-Infectious Diagnosis				
Lung Cancer	1.39 (0.36–5.36)	0.63	1.84* (0.42–8.10)	0.42
COPD	2.84 (1.06–7.59)	0.04	2.92 (1.02–8.38)	0.05
Any non-infectious diagnosis	2.09 (0.94–4.64)	0.07	1.95 (0.80–4.74)	0.14
**MACS HAART era**	OR (95% CI)	p-value	OR (95% CI)	p-value
Infectious Diagnosis				
Bacterial pneumonia	3.78 (2.35–6.10)	<0.001	4.14 (2.43–7.08)	<0.001
Acute sinusitis	1.59 (1.38–1.83)	<0.001	1.46 (1.28–1.68)	<0.001
Acute bronchitis	1.60 (1.32–1.94)	<0.001	1.51 (1.24–1.84)	<0.001
Any infectious diagnosis	1.60 (1.43–1.80)	<0.001	1.50 (1.34–1.68)	<0.001
	HR (95% CI)	p-value	HR (95% CI)	p-value
Non-Infectious Diagnosis				
Lung Cancer	3.48 (0.40–30.3)	0.26	2.65* (0.29–24.4)	0.39
COPD	1.16 (0.29–4.68)	0.84	1.61@ (0.36–7.19)	0.53
Any non-infectious diagnosis	1.53 (0.48–4.93)	0.47	1.68@ (0.49–5.72)	0.41
**WIHS HAART era**	OR (95% CI)	p-value	OR (95% CI)	p-value
Infectious Diagnosis				
Bacterial pneumonia	13.5 (4.3–42.6)	<0.001	9.55 (2.93–31.1)	<0.001
Acute sinusitis	2.27 (1.89–2.73)	<0.001	2.17 (1.79–2.63)	<0.001
Acute bronchitis	1.47 (0.83–2.63)	0.19	1.46 (0.79–2.68)	0.22
Any infectious diagnosis	2.37 (1.97–2.83)	<0.001	2.22 (1.84–2.68)	<0.001
	HR (95% CI)	p-value	HR (95% CI)	p-value
Non-Infectious Diagnosis				
Lung Cancer	1.63 (0.47–5.64)	0.44	2.45 (0.55–10.9)	0.24
Asthma	0.90 (0.74–1.11)	0.32	1.01 (0.87–1.20)	0.82
Any non-infectious diagnosis	1.17 (0.85–1.61)	0.33	1.28 (0.88–1.86)	0.19

Adjusted models include the variables age, race, cumulative pack-years smoking, alcohol use, and intravenous drug use unless otherwise noted.

* Adjusted for alcohol use, smoke years and age.

@ Excluded IDU due to large missing values.

**Table 4:** Incidence and person years for pulmonary disease.

		HIV-uninfected		HIV-infected	
		Incidence	IR	Incidence	IR
**MACS Pre-HAART**					
Infectious diagnoses					
	PCP	0	–	554	30.59
	NTM	0	–	4	0.22
	TB	0	–	9	0.5
	Bacterial pneumonia	9	0.41	150	8.28
	Acute sinusitis	1414	65.04	1359	75.05
	Acute bronchitis	544	25.02	650	35.89
	Any infectious diagnosis*	1753	80.64	2357	130.16
					
Non-infectious diagnoses					
	Lung Cancer	4	0.18	5	0.28
	COPD	6	0.28	11	0.61
	Any non-infectious diagnosis	10	0.46	16	0.88
**MACS HAART era**					
Infectious diagnoses					
	PCP	0	–	43	2.51
	NTM	0	–	0	–
	TB	0	–	0	–
	Bacterial pneumonia	28	2.31	108	6.29
	Acute sinusitis	1495	123.3	2596	151.24
	Acute bronchitis	579	47.75	1047	61
	Any infectious diagnosis*	1894	156.21	3312	192.95
Non-infectious diagnoses					
	Lung Cancer	1	0.08	5	0.29
	COPD	4	0.33	6	0.35
	Any non-infectious diagnosis	5	0.41	10	0.58
**WIHS HAART era**					
Infectious diagnoses					
	PCP	0	–	64	3.5
	NTM	0	–	28	1.53
	TB	0	–	15	0.82
	Bacterial pneumonia	5	0.8	120	6.56
	Acute sinusitis	804	135.2	4992	273.1
	Acute bronchitis	16	2.69	70	3.83
	Any infectious diagnosis**	807	135.7	5216	285.2
Non-infectious diagnoses					
	Lung Cancer	3	0.5	15	0.82
	Asthma	124	22.21	367	21.31
	Any non-infectious diagnosis	126	22.61	379	22.01

IR is per 1,000 person-years.

PCP = Pneumocystis pneumonia, NTM = non-tuberculosis mycobacterial infection, TB = pulmonary tuberculosis, COPD = chronic obstructive pulmonary disease.

* includes PCP, bacterial pneumonia, NTM, TB, bronchitis, and sinusitis.

** Includes PCP, bacterial pneumonia, NTM, TB, and sinusitis (bronchitis not included in WIHS analyses).

HIV-infected individuals in MACS continued to have a greater risk for incident respiratory infections compared to HIV-uninfected participants in the HAART era (**Table 3**), but much less than in the pre-HAART era. There were 2.51 cases of PCP per 1,000 person-years in HIV-infected participants (**Table 4**), and there were no cases of NTM or TB in either group. HIV-infected participants remained more likely to have bacterial pneumonia, acute sinusitis, acute bronchitis, or any infectious respiratory disease.

HIV-infected individuals in WIHS had more incident respiratory infections compared to HIV-uninfected participants (**Table 3**). There were 3.50 cases of PCP, 1.53 cases of NTM, and 0.82 cases of TB per 1,000 person-years in HIV-infected participants compared to no cases of these diseases for HIV-uninfected participants (**Table 4**). HIV-infected participants had increased rates of bacterial pneumonia, acute sinusitis, or any infectious respiratory disease.

### Non-infectious Pulmonary Diseases

Incident non-infectious lung diseases were more common in the HIV-infected MACS participants prior to HAART availability (**Table 3**). There was no significant difference in the risk for lung cancer, but in HIV-infected participants there was an increased risk for COPD compared to HIV-uninfected participants.

During the HAART era, HIV-infected individuals in MACS had no significant increased risk of COPD or lung cancer compared to HIV-uninfected participants (**Table 3**). There was one incident lung cancer recorded in the HIV-uninfected group, while in the HIV-infected group there were five cases.

In the WIHS cohort during the HAART era, there was not a significant effect of HIV status on incident non-infectious respiratory diagnoses (**Table 3**). HIV-infected participants had a greater lung cancer rate, but this difference was not statistically significant.

### Mortality

**Figure 1:**
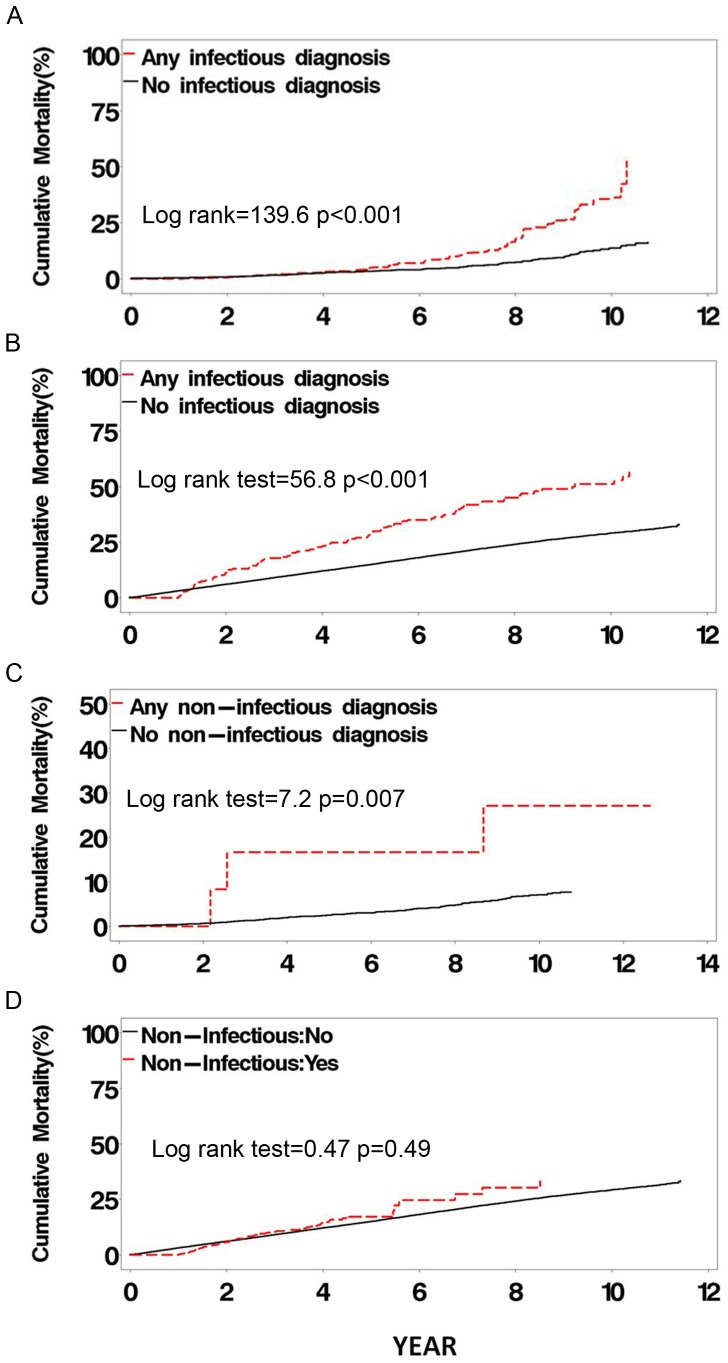
Unadjusted cumulative HAART era mortality. Unadjusted Kaplan-Meier mortality curves starting during the HAART era for HIV-infected participants who had any infectious respiratory disease vs. those who never had any infectious disease in the MACS (A) and WIHS (B) cohorts and for HIV-infected participants who had any non-infectious respiratory disease vs. those who never had any non-infectious disease in the MACS (C) and WIHS (D) cohorts. Time zero represents the start of the HAART era or seroconversion for participants who seroconverted during the HAART era.

During the HAART era, having respiratory disease was associated with an increased risk of all-cause mortality. Having any infectious respiratory disease was associated with increased mortality in MACS HIV-infected participants (adjusted-HR, 1.5; p<0.001) (**Figure 1a**) and HIV-uninfected participants (adjusted-HR, 1.8; p = 0.002) and WIHS HIV-infected participants (adjusted-HR, 1.9; p<0.001) (**Figure 1b**) and HIV-uninfected participants (adjusted-HR, 6.3; p<0.001). Having any non-infectious respiratory disease was associated with mortality in MACS HIV-infected participants (adjusted-HR, 3.4; p = 0.04) (**Figure 1c** **)** and HIV-uninfected participants (adjusted-HR, 7.6; p = 0.04) and WIHS HIV-uninfected participants (adjusted-HR, 1.9; p = 0.04), but not in WIHS HIV-infected participants **(** **Figure 1d**).

## Discussion

This study is the first to report the associations of HIV infection and respiratory diseases both before and after HAART availability and to assess the impact of respiratory disease on mortality during the HAART era. We tracked incidence of multiple infectious and non-infectious respiratory conditions in two large cohorts of HIV-infected and HIV-uninfected participants over a 20-year period. Both prior to and during the HAART era, infectious respiratory diseases such as PCP, bacterial pneumonia, and acute bronchitis were more frequent in HIV-infected compared to HIV-uninfected men and women. Prior to HAART, HIV-infected men were more likely to have COPD than uninfected men. In contrast, HIV was not an independent risk factor for non-infectious diagnoses in men or women after the introduction of HAART. Infectious respiratory conditions in HIV-infected individuals were associated with an increased risk of death whether sinusitis was included or not, while for non-infectious conditions this was true only in men. Although respiratory infections were also associated with increased mortality in the HIV-uninfected population, the higher incidence of respiratory infections in HIV-infected people suggests that improved prevention of infectious respiratory complications could be particularly important in the HIV-infected population.

Our findings mirror findings in the PCHIS and in the HAART era in the Veterans Aging Cohort study (VACS) [3], [6], [23]. Respiratory diseases with the highest incidence in HIV-infected individuals in PCHIS and MACS during the pre-HAART era were acute sinusitis, acute bronchitis, PCP, and bacterial pneumonia; and HIV was an independent risk factor for these infections in both cohorts. Similar to the VACS, during the HAART era HIV infection was still an independent risk factor for bacterial pneumonia, PCP, and TB [6]. It should be noted that TB incidence was quite low in these cohorts and may not relate to areas where TB is more common such as Africa and Asia.

COPD was increased in MACS HIV-infected compared to HIV-uninfected participants before HAART, but HIV infection was not an independent risk factor for non-infectious respiratory diseases in MACS or WIHS during the HAART era. The VACS found HIV infection to be a risk factor for non-infectious respiratory disorders such as COPD, lung cancer, pulmonary hypertension, and pulmonary fibrosis [6]. We did not find that HIV infection was significantly associated with lung cancer, similar to prior publications in the MACS/WIHS cohorts. [10], [24] This conflict may be due to non-infectious diagnoses of COPD and asthma in the present study were based on self-report, and use of self-report could potentially bias the findings or underestimate the true prevalence of diseases such as COPD and asthma as we have previously reported that HIV-infected individuals with chronic respiratory symptoms often do not undergo formal pulmonary function testing that would establish these diagnoses [16]. Other differences in the studies such as ethnicity, HIV risk factors, socioeconomic status, or access to health care could also account for the findings.

Few data exist about the interaction between HIV and asthma risk. HIV infection was not found to be a risk factor for asthma in the HAART era either in WIHS in the current study or in the VACS, and these data were not available in MACS. Contrary to our findings, two studies in children that show an increase in asthma diagnosis or asthma-related inhaler use in HIV-infected children on HAART [25], [26]. This discrepancy may reflect differences in asthma between children and adults, other differences between the populations, or the use of self-reported diagnosis instead of pulmonary function testing as a recent study found a high prevalence of reversible airway obstruction in an HIV-infected population [27].

The association of HIV infection and lung cancer has not been clearly determined, and results of different studies are conflicting[6], [8]–[10], [28]–[36]. In the current study, we did not find a significant association between HIV infection and lung cancer in either cohort. Other studies from these cohorts have not found HIV infection to be significantly associated with lung cancer, though the absolute number of lung cancers is quite small, limiting the power to detect a true association [10], [24]. However, studies from VACS show HIV infection to be independently associated with lung cancer when controlling for confounders such as smoking [6], [36]. However, in both the MACS [24] and the VACS, smoking was the greatest risk factor for lung cancer in both HIV-infected and HIV-uninfected participants, highlighting the importance of smoking avoidance.

We found that infectious respiratory diseases were independently associated with increased mortality during the HAART era. These findings are consistent with other reports that show increased mortality in HIV-infected persons related to lung infections [7]. Although increased risk of mortality in persons with respiratory diseases was also seen in HIV-uninfected participants, risk in HIV represents an important public health issue given the greater incidence of these infections in HIV. We found an association of non-infectious diagnoses with mortality in HIV in the MACS participants, but not WIHS. Other studies have found increased mortality from lung cancer in HIV-infected persons[37]–[39]. Additionally, the increased incidence of COPD in MACS pre-HAART may have contributed to increased mortality post-HAART, while we do not know the incidence of COPD in women. It is also possible that we did not see such an association because numbers of lung cancer cases were low.

Smoking is an important risk factor for lung diseases such as pneumonia, chronic obstructive pulmonary disease, and lung cancer, [40], [41] and smoking is important in the HIV-infected population because of the prevalence of smoking. In these cohorts in the HAART era, nearly 70–74% of the participants were current or former smokers, and cumulative pack-years smoked were greater in the HIV-infected participants. In light of these issues, we took care to control for smoking in our models assessing the association of HIV status and lung disease.

This study has several strengths compared to previous work examining respiratory complications of HIV. First, it includes both men and women and spans almost the entire course of the AIDS epidemic. The cohorts analyzed allowed the study of the effects of HIV infection on respiratory disease in a substantial number of women, minorities, and illicit drug users, reflecting the current HIV-infected population in the United States [17], [42]. Second, although there were differences between the HIV-infected and HIV-uninfected groups, the uninfected groups included similar populations of at-risk persons [18], [19]. Finally, we investigated a wide range of respiratory disorders and the impact of these disorders on mortality.

This study has several limitations. The MACS cohort may not reflect the current HIV epidemic in that it includes HIV-infected participants who were followed prior to HAART and received non-HAART antiretroviral regimens for a substantial period of time. MACS may not represent the HIV population in other parts of the world such as Africa where men who have sex with men is not as common a mode of HIV transmission [43]. The low prevalence of a viral load response to treatment in the WIHS cohort may not reflect the entire HIV-population as viral load response in other cohorts is close to 80–90% [44]. Diagnoses of COPD, asthma, sinusitis, and bronchitis were self-reported and not confirmed by specific criteria or testing in both groups which may result in underreporting and the incidence rates reported may not reflect the true incidence of these diseases in this population. It is difficult to assess whether there might have been more underdiagnosis in those with HIV infection or a greater chance for diagnosis as a result of HIV-infected persons coming in contact with medical care more often than HIV-uninfected persons. In addition, influenza may be an important infection related to lung disease in this cohort, but we did not have reliable data to accurately determine the influence of influenza. We did not evaluate other risk factors for infection like diabetes, renal disease, or other comorbidities that may also influence the risk for infections. Underdiagnosis of COPD is common in the general population but may be more common in those with HIV given decreased recognition of smoking behavior, lack of confidence in smoking cessation counseling among primary care providers of HIV-infected patients, and underutilization of pulmonary function testing in HIV[6], [16], [45]–[51]. We were unable to evaluate incidence of important conditions such as interstitial lung disease and bronchiectasis, data not collected in either MACS or WIHS. We chose not to determine the effect of duration or type of HAART on specific respiratory disease, and we considered HAART in the context of overall HIV care. We may have lacked the power to detect differences in incidence of the less frequent conditions including lung cancer and COPD. Also, in general, outcomes were too few to stratify the analysis by time period or different levels of treatment response to see how the effect of HIV-infection has changed over time during the HAART era or in participants with a good response to HAART. Given the relatively young age of the cohorts during the HAART era, a longer follow-up period may be needed to see any effect on these conditions. Therefore, we cannot exclude the possibility that HIV infection is an independent risk for COPD and lung cancer.

In conclusion, infectious and non-infectious respiratory diseases remained frequent morbidities of HIV infection throughout the course of the AIDS epidemic in both men and women. As in the HIV-uninfected population, respiratory diseases increased the risk of death in those with HIV. The specific contribution of HIV to the pathogenesis of non-infectious respiratory diseases remains to be determined, and interventions to treat or prevent both infectious and non-infectious respiratory diseases in people living with HIV could have an impact on health-related outcomes.
